# The market trend analysis and prospects of scaffolds for stem cells

**DOI:** 10.1186/2055-7124-18-11

**Published:** 2014-09-01

**Authors:** Seou Lee, Taehoon Kwon, Eun Kyung Chung, Joon Woo Lee

**Affiliations:** Techbiz, Room No. 903, 26 Seochojoongang-Ro, Seocho-Gu, Seoul, 137-918 Korea; KISTI, 66 Heogi-ro, Dongdeamoon-gu, Seoul, 130-741 Korea

**Keywords:** Regenerative medicine, Stem cell, Tissue engineering, Scaffold, Market analysis

## Abstract

**Background:**

Scaffolds are one of the three most important elements constituting the basic concept of regenerative medicine, and are included in the core technology of regenerative medicine along with stem cells and tissue engineering. Stem cells are very important technology because they are directly responsible for the regenerative treatment of the disease and the damaged tissue, but with regards to the technology and the products that use stem cells exclusively, there is a technical limitation of limited survival rate and the engraftment rate of the transplanted cell, and rather than recovering the damaged tissue fundamentally, there is a limit that the concept is more of just another medicine treatment using cells.

A scaffold is a natural or synthetic biocompatible material transplanted into a human body to be used as the exclusive treatment or as an assisted method of another treatment of a disease and for the recovery of damaged tissue. Therefore, according to the characteristics of the tissue to be applied, scaffolds must have the characteristics such as the excellent biocompatibility, biodegradability, minimum immunity and inflammation, proper mechanical strength and interaction between the material and the cells.

**Results:**

The world stem cell market was approximately 2.715 billion dollars in 2010, and with a growth rate of 16.8% annually, a market of 6.877 billion dollars will be formed in 2016. From 2017, the expected annual growth rate is 10.6%, which would expand the market to 11.38 billion dollars by 2021. Meanwhile, the world scaffold element technology market was approximately 4.57 million dollars in 2013, and by increasing 13.4% annually, it is estimated to expand to 10.63 million dollars by 2020. The Korean scaffold element technology market was about 22 million dollars in 2013, and with a steady growth of approximately 13.4% every year, it is prospected to be about 52 million dollars by 2020.

**Conclusions:**

In comparison to the medical material and medicine sales growth rate, the future scaffold element technology market is judged to be higher in growth possibility.

## Background

In the modern society, due to developments in technology and industry, there are increasing cases of defunctionalization or damage to tissues or organs from various accidents, diseases, and aging, and as the human body reaches its limits in self-regeneration ability, the need for proper and effective treatment methods is increasing rapidly [1–3].

For the treatment of damaged tissue or organs, organ transplantation from donors or animals is often attempted, but there are many problems such as a lack of organ donors, immune rejection response, and animal-derived viruses [4–8]. Therefore, the tissue engineering approach of using biomaterials to effectively substitute for or transplant a replacement of damaged biological tissues or organs while avoiding these problems is coming to the fore, and thus there is a need for various studies on these research topics [8–12].

Accordingly, studies on biomaterials useful in tissue regeneration are actively being conducted to design materials that can induce the regeneration of the damaged tissue or organ. Research is also currently being done on stem cell differentiation within scaffolds and mechanisms of the tissue regeneration on transplant to the human body and efforts on the development and application of its therapeutic method [13]. However, it is very difficult to form three-dimensional artificial organ similar to the structurally complex tissue within the human body due to the technical limits in the biomaterial development.

## Methods

Data for this research were gathered from primary and secondary sources as well as the databases of the Korea Institute of Science Technology Information. The key areas of the research process are described below.

### Primary research

Primary research sources relied for the databases of Korea Institute of Science Technology Information, past industry research service/study, economic and demographic data, and trade and industry journals. This research was conducted to map and analyze market and technology trends.

### Secondary research

Secondary research was used to supplement and complement the primary research. Interviews were conducted with physicians and surgeons from the key hospitals and senior sale/marketing managers from cell therapy product suppliers in South Korea.

## Results and discussion

### Concept and characteristics of scaffolds

Scaffolds are one of the three most important elements constituting the basic concept of regenerative medicine, and their role is adhesion and differentiation of the cells disseminated inside and outside of their structure, and the basis for providing the proper environment for cell proliferation and differentiation moving from the surroundings of the tissue and for providing the tissue form required [14]. Also, scaffold technology is included in the core technology of regenerative medicine along with stem cells and tissue engineering.

Stem cells are the most important technology because they directly take charge of regenerative therapy on the disease and the damaged tissue [15], but it is not easy to realize regenerative medicine’s goal of “fundamental recovery of the damaged tissue and organ” with stem cells alone [16]. Currently, various types of stem cells such as autologous and allogeneic adult stem cells, embryonic stem cells (ESCs), and induced pluripotent stem cells are being used in many attempts to cure disease, and for the adult stem cells, some products have already been developed for commercialization. However, technology and products using stem cells exclusively have technical limitations due to the limited survival rate and engraftment rate of the transplant cell, and with these limits, stem cell treatment become more of a concept of another medicine treatment using cells rather than a way to recover the damaged tissue fundamentally. In an attempt to overcome this technical limit, biomaterial technology for the transplant and delivery of stem cells and tissue engineering technology for engrafting biomaterials are being actively studied.

The scaffold is a natural or synthetic biocompatible material transplanted into the human body for the treatment of the disease and recovery of the damaged tissue, used exclusively or as a means of assistance to another treatment [14, 17]. Therefore, according to the excellent biocompatibility, biodegradability, minimum immunity and inflammation, proper mechanical strength, interaction between the material and the cell, interconnected porosity structure, and the characteristics of the tissue to be applied, characteristics such as the proper mechanical strength are required [18].

Currently, the biomaterials used clinically for the transplant of stem cells are natural biomaterials such as collagen, fibrin, chitosan, keratin, peptide, hyaluronate, hydrogel, silk protein, hydroxyl, and tri-calcium phosphate as well as approved medical macromolecules with biocompatibility with synthetic biomaterials such as PLA, PGA, PLGA, and PLC [18–22]. Natural biomaterials have excellent bio-functionality such as biocompatibility and biodegradability but have the disadvantages of limited mechanical and physical strength and low processability. On the other hand, synthetic biomaterials can be controlled the characteristics of their excellent mechanical property, flexible processability. Among the synthetic biomaterials, biodegradable biomaterials that self-degrade after inducing the regeneration and recovery of tissue in the human body are being studied greatly.

Researchers in tissue engineering technology have been mounting stem cells on biomaterials and cultivating them inside or outside the human body to produce artificial organs similar in structure and function to actual human tissues or organs.

Regarding current status of the biomaterial development technology in Korea, there are companies developing and producing single products such as badges. Most Korean biomaterial companies are focused on cell therapy products using cells rather than on producing primary cultured cells for studies or stem cells R&D. However, in other parts of the world, many companies are developing and commercializing cells and cultured elements such as primary cultured cells for study, stem cells, culture mediums, and coatings.

### Characteristics of the stem cell technology industry

Scaffold technology is applied as the element in the regenerative medicine field, relevant to the stem cell industry and also to the regenerative medicine industry by extension. Regenerative medicine is a high-tech convergence technology field of regenerating or substituting for a tissue and organ that is damaged or malfunctioning due to aging, disease, and an accident. Also, regenerative medicine is emerging as an alternative to treating intractable diseases such as dementia, diabetes, and spinal cord injury for which no proper therapy method currently exist, and it is being applied in various fields such as customized cell therapy products, biological tissues, and in bio-organ development. Regenerative medicine is receiving attention as a new growth engine to create enormous economic added value.

When we look at the main characteristics of the regenerative medicine technology and industry, it first becomes apparent that regenerative medicine technology mainly focuses on intractable and chronic diseases; therefore, there is a great demand for clinical treatment. Second, since unlike in the existing drug therapies, the subject is a living cell, there are technical and procedural difficulties in the process of approval. Third, because it is mostly transplanted through surgical processes, optimization and standardization of the clinical protocol and new medical technology and improvement in the health insurance system are required. Lastly, verification of clinical safety and effectiveness through long-term observation is very important.

The stem cell industry is a field which will likely develop in various areas such as cell therapy products, new drug developments, and bio-tissue engineering, which will be used widely in disease treatment. Since it is a new field that has not yet established its overall technology system, various types of research and development are being attempted for industrialization and performance creation. Due to the lack of R&D manpower and low technology maturity in the stem cell industry, there are many technical difficulties to overcome before actual commercialization can occur, but depending on future R&D efforts, the growth potential is high, and the market entry barrier is relatively low, which means that it is a field with high development possibility. Also, it is a high value-added application field that is based on biotechnology and converged with high technology such as IT, NT, etc. The industry not only has added-value on the medicine itself based on regenerative effects, but it also has front and back linkage effects with the medical industry, and high quality employment can be created with the advanced country type knowledge-based technology. Due to the long development period and high risk in the industrialization process, it is also a field that is generalized by government-led R&D support worldwide (Table 1).

**Table 1:** **Technology field and contents related to stem cells** [23]

Technology field	Contents
Bio-tissue engineering and engineering of bio-organs using stem cells	- The regenerative ability of the stem cell is used to culture the stem cell and the supporting cells, and the widely-differentiated tissue and organ regenerates the damaged tissue or organ to enable the treatment of the disease.
	- The bio-tissue using stem cell is high in biocompatibility and low in immune-rejection, expected to possess a big strength as the tissue engineering product.
Cell therapy product development using stem cells	- The stem cell extracted from the patient is differentiated/proliferated in a specific environment and injected into the relevant patient to substitute for the function of the damaged cell and tissue
	- The universality of the stem cell is expected to innovate in all medical fields of the cardiovascular system, the nervous system, the blood and immune system, the bone and cartilage system, and in the skin.
Efficiency of new drug development using stem cells	- Stem cells are differentiated into specific disease cells to discover candidate materials for new drugs, and the effectiveness verification is quickly performed in large quantities to improve the efficiency of new drug development.
	- During the clinical test process in new drug development, stem cells are differentiated into various cells and tissues, which are expected to verify the toxicity of the candidate material for the new drug more easily and quickly.
	- The cancer stem cell is the original material for anticancer drug development and for initial diagnostic technology development of cancer, prospected to be utilized in new anticancer drug and diagnostic agent development.

### Korean and world scaffold market size and prospects

Regarding the market for scaffolds, which are a biomaterial and one of the core element technologies in regenerative medicine, the scaffolds market data is very insufficient for clearly understanding and estimating its scope and size.

The element technology market for scaffolds has the characteristic of high relating effect with the front industry; therefore, the market trend, size, and prospects for scaffolds will be assumed to be similar to the trend of the front industry, the stem cell and regenerative medicine industry, and will be estimated using data from that industry.

Therefore, to estimate the Korean and world market size and prospects for scaffold element technology, the following data related to the stem cell market are considered.

First, total world market size, prospects, and growth rate of the stem cell field in which scaffolds are applied are identified. The world stem cell market was approximately 2.715 billion dollars in 2010 and with a 16.8% growth rate annually, the market will be 6.877 billion dollars in 2016 with an annual average growth of 10.6% starting in 2017, the market is prospected to expand to 11.38 billion dollars by 2021 [1] (Table 2).

**Table 2:** **World market analysis and prospects of stem cells** [1]

	2010	2011	2012	2013	2014	2015	2016	2017	2018	2019	2020	2021
Stem cells market ($m)	2,715	3,238	3,819	4,500	5,208	6,006	6,877	7,747	8,658	9,551	10,471	11,380
Annual growth (%)		19	18	18	16	15	14	13	12	10	10	9
CAGR (%)							16.8					10.6
Stem cells services ($m)	1,420	1,647	1,894	2,178	2,462	2,782	3,115	3,427	3,770	4,109	4,479	4,882
Annual growth (%)		16	15	15	13	13	12	10	10	9	9	9
CAGR (%)							14.0					9.4
Cord blood banking ($m)	1,120	1,344	1,572	1,824	2,061	2,288	2,517	2,743	2,963	3,200	3,424	3,629
Annual growth (%)		20	17	16	13	11	10	9	8	8	7	6
CAGR (%)							14.4					7.6
Stem cell therapies ($m)	125	194	296	439	623	872	1,177	1,507	1,854	2,169	2,494	2,794
Annual growth (%)		55	53	48	42	40	35	28	23	17	15	12
CAGR (%)							45.3					18.9
Bone marrow transplants ($m)	50	53	56	59	62	64	67	70	72	73	75	75
Annual growth (%)		6	6	5	5	4	4	4	3	2	2	1
CAGR (%)							5.0					2.4

Second, in the stem cell market, the market size of which scaffold element technology is included is assumed. As we can see in Table 3, the element technology field, which is classified into cell lines, cell culture fluid, and cell carriers, occupies an average of about 10.15% (2010 ~ 2015) of the total technology market related to cell treatment, and from 2005 to 2015, an annual average growth rate of 17.2% is prospected [24].

**Table 3:** **Cell treatment market size and prospects (unit: 100 million dollars)** [24]

Technology	2005	2010	2015	CAGR
Stem cells	20	52	109	18.5%
Cord blood	5	10	23	16.5%
Tissue engineering	69	135	232	12.9%
Transfusion products	128	224	350	10.6%
Cell-based gene therapy	15	30	59	14.7%
Capsule cell treatment	4	19	31	22.7%
Cell-based cancer vaccines	9	16	29	12.4%
Heteroplastids	6	19	32	18.2%
Element technology (Cell lines, cell culture fluid, cell carriers)	20	57	98	17.2%
Total	276	562	963	13.3%

**Figure 1:**
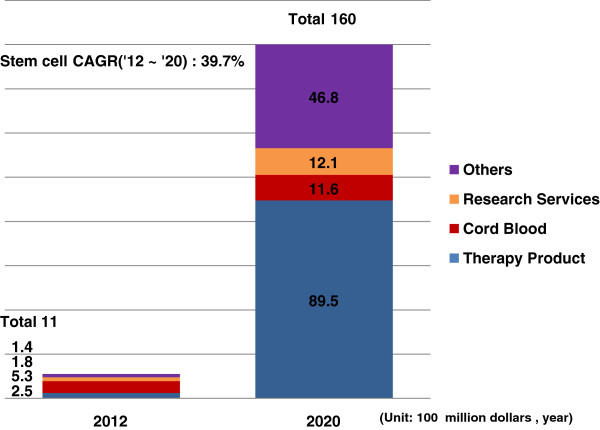
**World market prospects of stem cells** [25] **.**

Third, the world stem cell market is estimated to grow rapidly from 1.1 billion dollars in 2012 to 16 billion dollars by 2020 with an annual growth rate of 39.7%. Within this, the world market size of the cell culture and consumables market, including the scaffold development technology market, was 320 million dollars in 2012 and is prospected to expand to 420 million dollars in 2015. Also, in 2011, the Korean sales related to stem cells make up a small-sized market of approximately 54 billion won, estimated to include 40 billion won in cord blood storage and 14 billion won in medicine, and the market of the consumables and instrument sector in which scaffold technology is included is reported as not being formed [25].Through these results, we can estimate that the Korean stem cell market size is about 4.9% of the world market, and this percentage can also be applied to the scaffold element technology market (Figure 1).

If we use the data above to estimate the market size and prospects of the Korean and world scaffold element technology markets, the world scaffold element technology market was approximately 457 million dollars in 2013, and by growing 13.4% annually, the market will expand to 1.063 billion dollars in 2020. The Korean scaffold element technology market was 22 million dollars in 2013, and through a steady increase of 13.4% like the world annual average growth rate, it is prospected to be 52 million dollars in 2020. In the last three years, the sales growth rate of the same industry, medical materials and medicine was 6.3%, which is 2.2% higher than the 4.1% of the last three years’ average economic growth rate, and the average estimated sales growth rate for the next 5 years of the market in which the evaluation technology is included is 13.4%. As it exceeds the recent average economic growth rate of 4.1%, it is judged that the growth possibility of the scaffold element technology market is high (Table 4).

**Table 4:** **Korean and world scaffold element technology market size and prospects (unit: million dollars)**

Division	2012	2013	2014	2015	2016	2017	2018	2019	2020	CAGR (‘12~’20)
World stem cell market	3,819	4,500	5,208	6,006	6,877	7,747	8,658	9,551	10,471	13.4%
World scaffold element technology market (×10.15%)	388	457	529	610	698	786	879	969	1,063	
Korean scaffold element technology market (×4.9%)	19	22	26	30	34	39	43	48	52	

### Relevant industry trend

Though approximately 500 companies are participating in the tissue engineering field worldwide, these companies are very difficult to identify precisely due to frequent change in company names, mergers, and acquisitions. Of the approximately 500 companies, about 303 of them are located in the US, the UK, Germany, Japan, or Sweden. About 230 companies are closely related to regenerative medicine, and about 27 of these companies are focusing on the discovery and development of drugs using stem cells, while another 27 companies are focusing on application programs. Therefore, 148 companies are developing biomaterials such as scaffolds, showing that it is not a small number compared to the total number of tissue engineering companies.

Along with developing biomaterials, some companies are combining stem cells to develop tissue engineering products for each tissue. Seventy-three companies are investigating how to develop tissue engineering products. The areas of competitive activity are the US and the UK, and there is a severe variability between the companies in these areas. It is especially difficult to identify the relevant companies in the US due to changes in company names Generally, large public companies are participating in regenerative medicine, other companies are sufficiently performing the core activity, and some companies are devoting their efforts to developing target products in the new business field [18].

**Figure 2:**
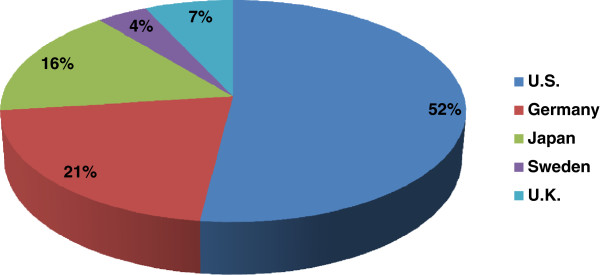
**Geographic distribution of tissue engineering firms in major markets** [18] **.**

Companies and research institutes related to tissue engineering share more knowledge and information and promote industrial development when they concentrated nationally or geographically to form a strong cluster. When we look at the geographic distribution of tissue engineering firms and research institutes (Figure 2), the US is the highest with 52%, followed by Germany (21%), Japan (16%), the UK (7%), and Sweden (4%) [18].

In Korea, there are no companies selling scaffold products, but there are R&D results. In 2012, The Korea University mechanical engineering department research team used micro-fluid technology to fix the extracellular matrix such as collagen to succeed in developing the technology of culturing various cells three-dimensionally.

**Figure 3:**
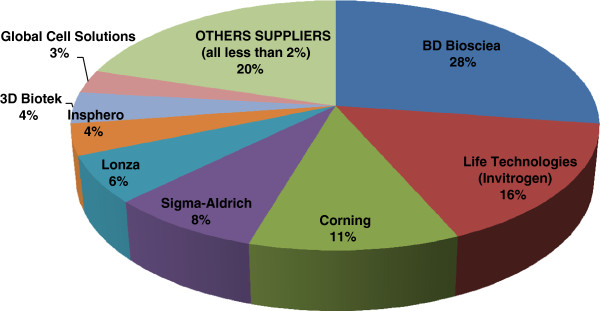
**Estimated supplier share of 3D consumables and instruments market** [26] **.**

In other parts of the world, there are many multinational companies related to three-dimensional cell culture, and among them, BD Biosciences has the highest awareness, followed by Life Technologies(Invitrogen), Corning, 3D Biotek, and Insphero.Also, among the companies related to three-dimensional cell culture consumables and instruments, BD Biosciences is the largest with a 27% market share, followed by Life Technologies(Invitrogen) (16%), Corning (11%), Sigma-Aldrich (8%), Lonza(6%), Insphero(4%), 3D Biotek(4%), and Global Cell Solutions (3%), showing that there are many companies competing (Table 5 and Figure 3).

**Table 5:** **Most purchased from suppliers of 3D cell culture consumables and instruments** [26]

Company	Primary supplier	Secondary supplier	% of all selections	Total no. of selections
3D Biomatrix	0%	3%	2%	2
3D Biotek	1%	6%	4%	5
BD Biosciences	41%	11%	27%	35
Bellbrook Labs	0%	2%	1%	1
CellASIC	3%	0%	2%	2
CELLnTEC	1%	3%	2%	3
Corning	9%	14%	11%	15
Electrospinning Company	0%	0%	0%	0
EMD-Millipore	3%	2%	2%	3
Global Cell Solutions	4%	2%	3%	4
GlycosanBiosystems	0%	2%	1%	1
Hamilton Company	0%	2%	1%	1
Insphero	4%	3%	4%	5
InvivoSciences	0%	2%	1%	1
Kuraray	0%	0%	0%	0
Life Technologies (Invitrogen)	16%	16%	16%	21
Lonza	3%	10%	6%	8
MatTek	3%	2%	2%	3
Microtissues	0%	0%	0%	0
OrganDot 3D	0%	0%	0%	0
QGel Bio	0%	0%	0%	0
RealBio Technology	0%	0%	0%	0
RegeneMed	1%	1%	1%	1
Reinnervate	1%	2%	2%	3
Scivax	3%	2%	2%	2
Sigma-Aldrich	4%	8%	8%	11
Synthecon	1%	2%	2%	2
TAP Biosystems	0%	1%	1%	1
Tissue Growth Technologies	0%	0%	0%	0
ThermoScientific	0%	2%	2%	2
Zyoxel	0%	0%	0%	0
Total	69	63	132	132

The following are world cell and tissue suppliers, represented by American Type Culture Collection(ATCC), Asterand plc, BioE, Life technologies, Cell Systems Biotechnologie Vertrieb GmbH, and Lonza Group Ltd., and they supply cell-based products for the cell therapy product and various human and animal cells or tissues (Table 6).

**Table 6:** **Selected suppliers of cells and tissues** [18]

Company	Product
American Type Culture Collection(ATCC)	Supplies many types of cells lines and bioproducts.
Asterand plc	Provides human biomaterials for drug testing(human primary cells and cell lines, RNA, tissue microarrays, frozen and fixed human tissues)
ATCC-LCG Promochem Partnership	Facilitates distribution of ATCC culture and bioproducts to life science researchers in Europe
BioE,	Multi-Lineage Progenitor cellTM, a human umbilical cord blood-derived, clonal, multipotent stem cell line; unique cell reagents.
Life Technologies	Keratinocytes and other epithelial cells, endothelial cells, fibroblasts, etc.
Cascade Biologics	Keratinocytes and other epithelial cells, endothelial cells, fibroblasts, etc.
Cell Systems Biotechnologie Vertrieb GmbH	Cell biology products including human primary cells, 3D skin cell systems, and cell models for angiogenesis, blood-barrier, and inhalation toxicology.
Lonza Group Ltd.	Primary human and animal cells with customized media systems, Clonetics® and Poietics® Cell Systems.
Musculoskeletal Transplant Foundation(MTF)	Pieces of human bone, skin, and other tissues; FlexHD, an acellular dermal matrix, primarily for surgery.
National Cancer Institute(NCI)	Tissues, cell lines, databases, arrays, etc. primarily for cancer research.
National Cell Culture Center(NCCC)	Division of Biovest International; provides customized, large-scale cell culture services.
National Disease Research Interchange(NDRI)	Human tissues, organs, and derivatives for research.
National Stem Cell Bank(NSCB) at WiCell	Established to acquire, characterize and distribute the 21 human embryonic stem cell lines and their sub-clones to federally funded research programs; they currently have 13 of the 21 cell lines in the federal registry.
NIH Human Embryonic Stem Cell Registry	Lists the derivations of stem cells that are eligible for federal funding and provides contact information for acquiring the cell lines.
OnCore UK	Human specimens for cancer research
ProBioGen AG	Specializes in mammalian cell engineering and cell culture: design of custom immortalized cell lines for vaccine and protein production, or for other customer needs.
SciKon Innovation, Inc.	Human matrix cell culture surface; human stellates; human hepatocyte cryopriserved cells.
Tebu-bio laboratories	Various cryopreserved primary cells; cell biology services such as cell amplification, differentiation, transfection, mycoplasma testing; establish cellular models overexpressing of silencing a protein of interest.
Zen Bio	Human adult stem cells, preadipocytes, adipocytes, primary human hepatocytes; specialized media.

The front industry companies of the scaffold market for stem cells will be specialized companies, institutions and general hospitals developing cell therapy products. Korean cell-therapy-product-related companies are Anterogen, Medipost, Cha Biotech, Pharmicell, SewonCellontech, and Tegoscience. Table 7 shows the Korean companies who developed cell therapy product approved by the FDA in US as well as their products. Also, 44 hospitals including Seoul ASAN Medical Center, Severance Hospital, Samsung Medical Center, Seoul National University Hospital, and Seoul St. Mary’s Hospital were investigated to be specialized hospitals [27].

**Table 7:** **Cell therapy products approved in South Korea** [18]

Company	Product	Indication
Anterogen	Adipocel	Adipose stem cell
	Cupistem®	Adipose stem cell
	Queencell®	Autologous Mesenchymal stem cell
Sewon Cellontech	RMS ossron	Cartilage cell
	Chondron	Bone stem cell
Chabiotech	Hyalograft-3D+	Autologous skin fibroblasts
	Autostem	Adipose cell
Creagene	Creavax-RCC+	Dendritic cell
FCB-Pharmicell	Heartcellgram-AMI	Autologous Bone Marrow-Derived Mesenchymal stem cell
Green Cross cell	Immuncell-LC®	Adjuvant therapy for patients whose tumor has been removed after curative resection for Hepatocellular Carcinoma
MCTT	KERAHEAL^TM^	Human epidermal keratinocyte
Medipost	Cartistem®	Human umcilical cord blood-derived mesenchymal stem cell product for cartilage regeneration
S-Bio Medics	Cureskin	Autologous fibroblast therapy
Tegoscience	Holoderm®	Epidermal cell
	Kaloderm®	Epidermal cell

Around the world, there are many major companies developing stem cell products such as Osiris Therapeutics, NuVasive, Aastrom Biosciences, BrainStorm Cell Therapeutics, Genzyme, International Stem Cell, Medcell Biosciences, Medistem, Opexa Therapeutics, Plureon, Revicor and StemCyte. Many of them are forming partnerships with pharmaceutical companies, and most of them are known to be producing products for the treatment of burns, wounds and skeletal defects (Table 8).

**Table 8:** **Leading commercial cell therapy products and companies in 2012** [18]

Company	Product	Indication
Advanced Biohealing, a Shire Company	Dermagraft	Diavetic foot ulcers
Allosource distributed by NuVasive	Osteocel	Skeletal defects
Alphatec Spine	PureGen	Musculoskeletal defects
Altrika	Myskin	Wounds
Altrika	Cryoskin	Wounds, burns
AvitaMadical	ReCell	Wounds, burns, scars
BioDlogics(distributed by America)	BioDfentor	Wound healing, soft tissue defects, skeletal defects
BioDlogics(distributed by America)	BioDfence	Wound healing, soft tissue defects, skeletal defects
Dendreon	Provenge	Prostate cancer
Fibrocell	laVin	Nasolabial fold wrinkles(smile lines)
Genzyme(a Sanofi company)	Carticel	Articular cartilage repair
Genzyme(a Sanofi company)	Epicel	Severe burn and skin replacement
Genzyme(a Sanofi company)	MACI	Articular cartilage repair
NuTech	NuCel	Wound healing, soft tissue defects, skeletal defects
Organogenesis	Appligraf	Venous leg ulcers, diabetic foot ulcers
Organogenesis	GINTUIT	Gingival and alveolar mucosal surface defects
Orthofix	Trinity Evolution	Musculoskeletal defects
Osiris Therapeutics	Grafix	Acute and chronic wounds
TiGencs	ChondroCelect	Articular cartilage repair
Zimmer Orthobiologics	DeNovo NT	Articular cartilage repair

## Conclusions

There can be differences in industrial prospects depending on the market and product scope of stem cells, but in most of the global market analysis report, rapid growth of the stem cell market is prospected.

Through the positive influences of unfulfilled medical markets for treatment for diseases such as leukemia and nervous system diseases and active stem cell research support by the government, stem cell bank services, and the activation of medical tourism, growth in stem-cell-related industries is expected. However, there are factors hindering the industrial activation such as high treatment cost and government regulation due to ethical concerns, but opportunity factors such as the continuous increase of neurodegenerative diseases such as Parkinson’s disease, the growth of emerging countries such as India and China, and the increase of availability on new drug development are prospected to lead to growth in the stem cell industry.

Therefore, the biomaterials included in the element technology of the stem cell and regenerative medicine industry, in other words, the scaffold market, is also a field expected to expand in its market together with the growth of the front industry. The market size of three-dimensional cell culture related consumables was estimated to grow at an annual average rate of 23.5% from 34 million dollars in 2011 to 64 million dollars in 2014, and it is prospected to grow strongly after 2014 [26].

Also, as results of the market research of industries related to stem cells and regenerative medicine to estimate the Korean and world scaffold market sizes, the 2013 world scaffold element technology market was 457 million dollars and expected to grow 13.4% annually, resulting in an expansion to 1.063 billion dollars in 2020. The Korean scaffold element technology market was about 22 million dollars in 2013 and is expected to have a steady growth of 13.4%, the same as the world annual average growth rate, which would result in a market size of 52 million dollars by 2020. By comparing with growth rate of medical materials and medicine sales, the future scaffold element technology market is judged to be higher in growth possibility.

The scaffold market is in the initial market stage of research and technology development progressing actively, and market is difficult to identify accurately due to the frequent M&A by the companies in the market and many problems that need to be overcome technologically, but many specialized companies and research institutes, both in Korea and in other parts of the world, are actively conducting research to develop scaffolds for regenerative medicine that can perform a similar role as the extracellular matrix, and policies and systems on stem cells and regenerative medicine are quickly expanding and being established cross-nationally for the quick commercialization of products. Therefore, the future prospects of the stem cells and regenerative medicine industry and other relevant industries are very bright.
